# Genome-Wide Analysis of the *DNA-Binding with One Finger* Gene Family Reveals Soybean Expression Pattern and Functional Analysis

**DOI:** 10.3390/ijms26136192

**Published:** 2025-06-27

**Authors:** Chuanjie Gou, Guyue Zhang, Ziyuan Deng, Chenyang Lin, Haiyang Li, Huan Liu, Xiaomei Fang

**Affiliations:** 1 Guangdong Provincial Key Laboratory of Plant Adaptation and Molecular Design, Innovative Center of Molecular Genetics and Evolution, School of Life Sciences, Guangzhou University, Guangzhou 510006, China; 2 College of Agronomy and Biotechnology, Southwest University, Chongqing 400716, China

**Keywords:** soybean, *Dof* gene family, phylogenetic analysis, expression patterns, salt stress

## Abstract

The Dof (DNA-binding with one finger) domain protein family is a plant-specific zinc finger transcription factor family that plays a role in various biological processes in plants. However, research on *Dof* transcription factors in soybean (*Glycine max*) remains limited. In this study, we identified 79 putative soybean *Dof* genes, which are distributed across the entire genome. A comparative phylogenetic analysis of the *Dof* gene family in soybean, *Arabidopsis*, rice, maize, and *Medicago* revealed five major groups. The synteny relationship analysis showed a large number of gene duplication events in soybean. Twelve cis-acting elements were detected in the promoter region of the *Dof* gene, including five hormone response elements and several environmental response elements. Expression pattern analysis indicated that most *Gmdof* genes exhibited specific expression patterns. Nine genes in group V, which exhibited higher expression in the root, were identified as significantly responsive to salt stress through qRT-PCR. The possible biological functions of several *Gmdof* genes were discussed, including *Gmdof11.2*, *Gmdof2.1*, and *Gmdof16.2*. In summary, this study integrated phylogenetic analysis with genome-wide expression profiling to provide valuable information for understanding the functional characteristics of *Dof* genes in soybean.

## 1. Introduction

Transcription factors, also known as sequence-specific DNA-binding factors, are key regulators of gene expression, which are capable of binding to specific cis-acting elements in the promoter regions of genes and activating or inhibiting the transcription of downstream target genes [1]. Plant TFDB V5.0 (https://planttfdb.gao-lab.org/index.php, accessed on 16 June 2025) reports that 6150 transcription factors are identified and classified into 57 families in soybean. Dof (DNA-binding with one finger) proteins belong to a plant-specific transcription factor (TF) gene family and are characterized by a Cys2/Cys2 (C2/C2)-type zinc finger domain at the N-terminal, and they bind specifically to cis-regulatory elements with a (T/A)AAAG core sequence [2,3,4,5]. With the whole-genome analysis of many species, an increasing number of Dof members have been discovered in different species, including 36 Dof genes in *Arabidopsis thaliana* [6], 30 in *Oryza sativa* [7], 18 in *Zea mays* [8], and 40 in *Medicago sativa* [9].

Dof transcription factors play a crucial regulatory role in various physiological and biochemical processes and exhibit functional diversity. In particular, Dof proteins participate as transcriptional regulators in seed development [10,11] and vascular development [12,13]. In addition, Dof transcription factors also contribute to the responses to various abiotic stresses in plants. Tomato SlDof22 [14], wheat Dof protein TaZNF [15], Arabidopsis AtDof5.8 [16], watermelon ClDof29 [17], alfalfa MsDof10, MsDof35, and MsDof39 [9] played an important role in the salt signaling pathway. As a Dof transcription factor, CDF has been reported to be widely involved in the responses to various abiotic stresses, such as Arabidopsis *cdf3-1* [18], tomato *SlCDF1* or *SlCDF3* [19], Brassica *BnCDF1* [20], cotton *GhDof1* [21], and rice *OsDof1* [22]. Recent research reported that Dof4.6 and XND1 jointly regulate root hydraulics and drought responses in *Arabidopsis* [23].

Although a considerable number of Dof transcription factors (TFs) have been reported in the model plant *Arabidopsis thaliana* and other species, the functions of most Dof family members remain unclear. This is particularly true for soybean (*Glycine max*), where functional characterization of Dof transcription factors is extremely limited. *GmDof4* and *GmDof11* had been reported to be involved in the lipid metabolism pathway of soybean. These DOF proteins can directly bind to cis-regulatory elements in the promoters of target genes, activating the expression of downstream genes involved in fatty acid biosynthesis, thereby regulating lipid metabolism at the transcriptional level [24]. Recently, *GmDof41*, as a Dof gene in soybean, has been identified as responsive to drought, salt stress, and exogenous ABA treatment. The transcription factor DOF41 directly binds to the promoter region of *GmDREB2A*, a gene encoding a DREB2-type protein implicated in regulating plant tolerance to abiotic stress [25]. Another study reported that Dof bound to the *PIF* promoter and regulated its expression. The regulatory network was speculated to improve the structure of soybean plants under shaded environments [26].

Since the soybean *Dof* gene plays a crucial role in many important developmental processes and responses to various environmental stresses, in-depth research on the soybean Dof family is of great significance. This study conducted a detailed analysis of the soybean *Dof* gene family, focusing on sequence phylogeny, duplication status, and expression profiles. The expression profiles of these genes with high expression in root were further explored for salt stress through RNA-seq data, providing insights into the potential specific functions of the predicted *GmDof* genes. These findings lay the foundation for further studies on the evolution and functional characteristics of the *Dof* gene family in soybean.

## 2. Results

### 2.1. Identification and Chromosomal Location of Dof Gene Family in Soybean

To identify the *Dof* gene family in the soybean genome, a BLAST search in TBtools-II v2.210 was conducted using the conserved Dof domain amino acid sequence against the Glycine max v1.2 genome (http://www.phytozome.net, accessed on 4 July 2024). A total of 79 non-redundant *Dof* transcription factor-encoding genes were identified across the entire genome (Supplementary Table S1). The predicted GmDof proteins contain the conserved Dof domain, confirming their membership in the Dof transcription factor family. These 79 soybean *Dof* genes were numbered from *Gmdof1.1* to *GmdofU* (the latter not being anchored to a chromosome in the annotation) according to the naming convention established for Arabidopsis, and classified based on their locations on different chromosomes. The peptide chain lengths of the identified *Gmdof* genes ranged from 147 to 555 amino acids, with an average length of 335 amino acids (Supplementary Table S1). The molecular weights of the proteins ranged from 9.16 (*Gmdof8.1*) to 54.67 (*Gmdof9.2*) KDa. For the subcellular localization, only 6 *Gmdofs* were located outside the nucleus (Supplementary Table S1).

**Figure 1:**
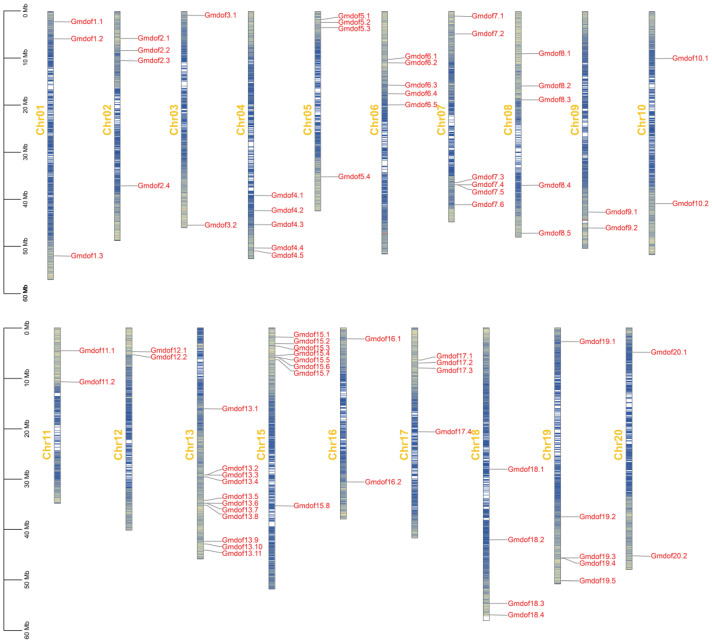
Chromosomal location of *Dof* gene family in soybean.

Genomic chromosomal localization analysis revealed that the *Gmdof* genes are non-randomly distributed across 19 chromosomes (Figure 1). Excluding the unassembled *GmdofU* gene, nearly all *Gmdof* genes are located on the chromosome arms, with no genes found in the heterochromatic regions surrounding centromeric repeat sequences. Among these chromosomes, chromosome 13 contains the most *Gmdof* genes and chromosome 14 contains no *Gmdof* genes. Several chromosomes display significant clustering of *Gmdof* genes, particularly those with higher gene density. For example, *Gmdof7.4* and *Gmdof7.5* are located within an 8.8 kb segment on chromosome 7, while *Gmdof15.5* and *Gmdof15.6* are found within a 19 kb segment on chromosome 15. Similarly, four genes on chromosome 13 (*Gmdof13.2* and *Gmdof13.3*, as well as *Gmdof13.6* and *Gmdof13.7*) are clustered into two groups, one spanning 10 kb and the other spanning 13 kb (Figure 1).

### 2.2. Phylogenetic Relationships and Gene Structure of Soybean Dof Genes

**Figure 2:**
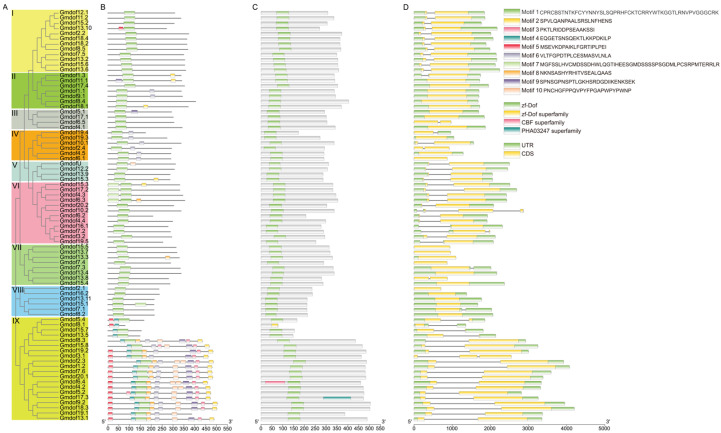
Evolutionary tree, conserved motif, structural domains, and gene structure of Dof gene family. (**A**) Evolutionary tree; (**B**) conserved motif; (**C**) structural domains; (**D**) gene structure.

To elucidate the functional differences among the various Dof genes, structural characteristics and conserved motifs were analyzed. A phylogenetic tree was constructed based on the full-length amino acid sequences of all Gmdof proteins, using a rooted tree approach (Figure 2A). The sequence similarity and topology of the phylogenetic tree categorized the soybean *Dof* gene family into nine subgroups (subgroups I–IX). Each subgroup contained 4–19 members, and the high bootstrap values within each subgroup indicated that these *Dof* genes share a common origin. Additionally, conserved motifs were identified using the MEME program (Figure 2B). The results revealed that these genes exhibit varying numbers, classifications, and positions of motifs among different genes. The number of conserved motifs in the *Dof* genes of soybean and wild soybean ranged from 1 to 10, with motif 1 being present in all *Dof* genes. Most of the *Dof* genes in subgroup IX contained 8–10 motifs. Furthermore, *Dof* genes within the same evolutionary branch displayed similar conserved motif patterns (Figure 2C). In the domain analysis, all *Dof* genes contained the zf-Dof domain, except Gmdof8.1, which had the zf-Dof superfamily domain. In addition, *Gmdof6.4* and *Gmdof17.3* had the CBF superfamily and PHA03247 superfamily domain, respectively.

It is well known that gene structure diversity may represent an evolutionary trend within gene families. To further explore the structural diversity of *Dof* genes, we compared the exon/intron structures of the *Dof* genes in soybean (Figure 2D). According to the predicted structures, 29 *Gmdof* genes lack introns, while 48 genes contain a single intron, typically located upstream of the Dof domain. Within the same subgroup, the most closely related members typically exhibit the same exon/intron pattern, and the position and length of introns are nearly completely conserved across most subgroups.

### 2.3. Phylogenetic Analysis of the Dof Gene Family in Soybean, Arabidopsis, Rice, Maize, and Medicago

**Figure 3:**
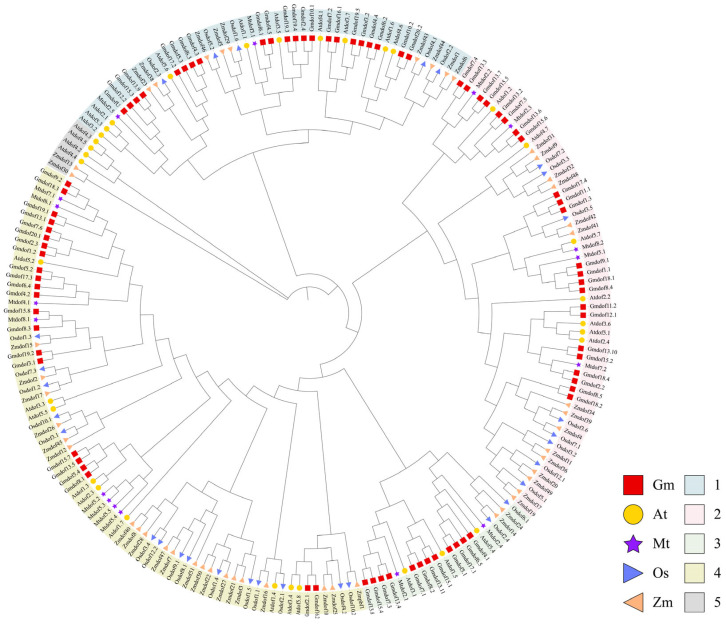
Phylogenetic analysis of the *Dof* gene family in soybean, Arabidopsis, rice, maize, and Medicago. The various colors on the periphery represent the different groups (or subgroups) of GmDof genes.

To investigate the molecular evolution and phylogenetic relationships of Dof domain proteins in soybean, Arabidopsis, rice, maize, and alfalfa, a multiple sequence alignment was conducted for the 79 predicted Gmdof proteins, 33 Arabidopsis Dof proteins, 30 rice Dof proteins, 49 maize Dof proteins, and 17 alfalfa Dof proteins. Based on the alignment results of all Dof amino acid sequences, a phylogenetic tree was constructed using the Maximum Likelihood (ML) method (Figure 3). The ML tree revealed that all Dof family proteins from the five plants were classified into five major clades, consistent with previous studies [8,13]. Four of these clades are the largest branches, comprising 75 members, which account for 36.1% of the total Dof genes. The remaining clades consist of 48 members (clade 1), 58 members (clade 2), 21 members (clade 3), and 6 members (clade 5). Overall, the majority of subfamilies exhibit interwoven distribution patterns, suggesting that the expansion of the *Dof* gene family occurred prior to the divergence of soybean, Arabidopsis, rice, maize, and Medicago.

### 2.4. Synteny Analysis of Gmdof Genes

**Figure 4:**
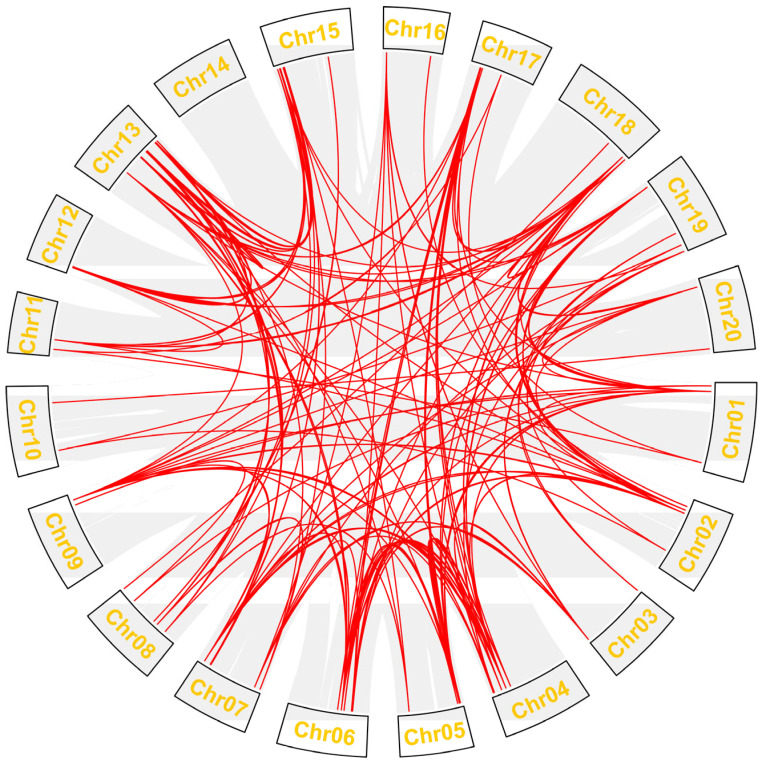
Schematic of the interchromosomal relationships of the Dof genes in soybean. The collinear blocks are shown in gray lines. The synteny *Dof* gene pairs are highlighted in red.

To assess the synteny of the *Gmdof* genes, we analyzed the 79 *Gmdof* genes in soybean to evaluate the syntenic relationships among the members of the soybean Dof family (Figure 4). Among the 79 *Gmdof* genes in soybean, a total of 284 gene duplication segments were identified. The 13th chromosome harbors the highest number of gene duplication segments, totaling 36, followed by the 4th, 7th, and 15th chromosomes, with 22 duplication segments. No gene duplication segments were found on the 14th chromosome, and no gene pairs were detected within the same chromosome. Overall, these identified *Gmdof* gene pairs provide strong evidence for the whole-genome duplication events in soybean.

### 2.5. Analysis of Cis-Acting Elements in the Promoter Region of Dof Genes

**Figure 5:**
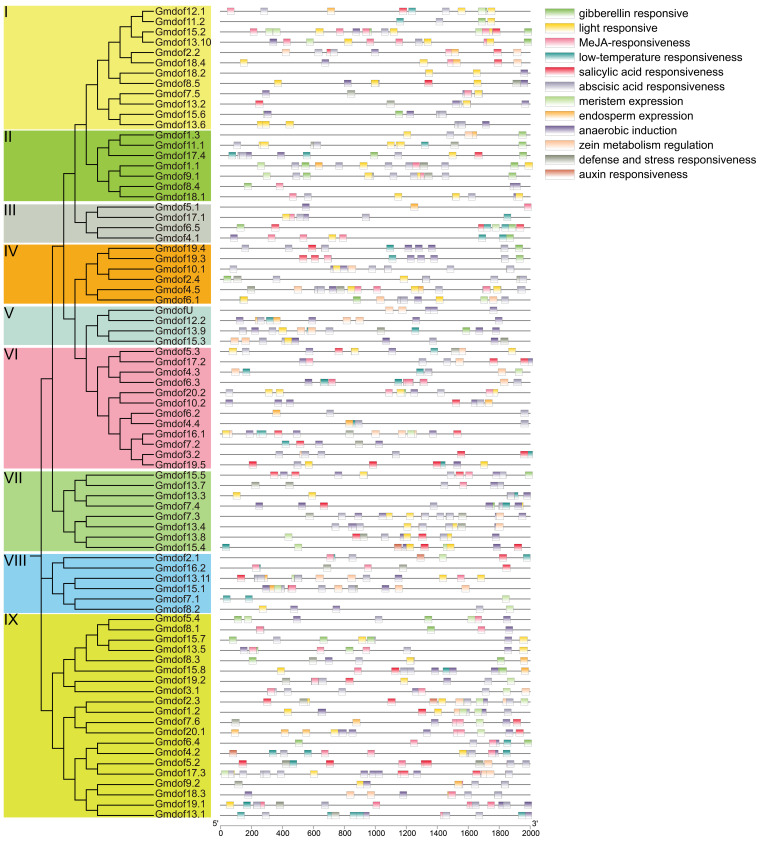
Analysis of cis-acting elements of the *Dof* gene family.

To better understand the function of *Dof* genes and the precise regulation of stress-responsive gene expression, the cis-acting elements in the promoter region of *Dof* genes were identified and analyzed (Figure 5). Twelve cis-acting elements were detected in the promoter region of the *Dof* gene, including five hormone response elements (gerber, jasmonic acid, salicylic acid, abscisic acid, auxin), as well as environmental response elements (such as light, low temperature, anaerobic, defense and stress), and development-related expression or regulatory elements (meristem, endosperm, and zein metabolism). A total of 867 elements were identified in promoter region of Dof gene families, and the most responsive elements were abscisic acid (213), followed by jasmonic acid (152), and anaerobic induction (122). There was no obvious distribution pattern of cis-acting elements of genes in each group. These abundant cis-acting elements also indicate that the *Dof* gene family is involved in a variety of plant regulatory pathways and has important significance for crop growth and development.

### 2.6. Expression Pattern of Dof Genes in Various Tissues

**Figure 6:**
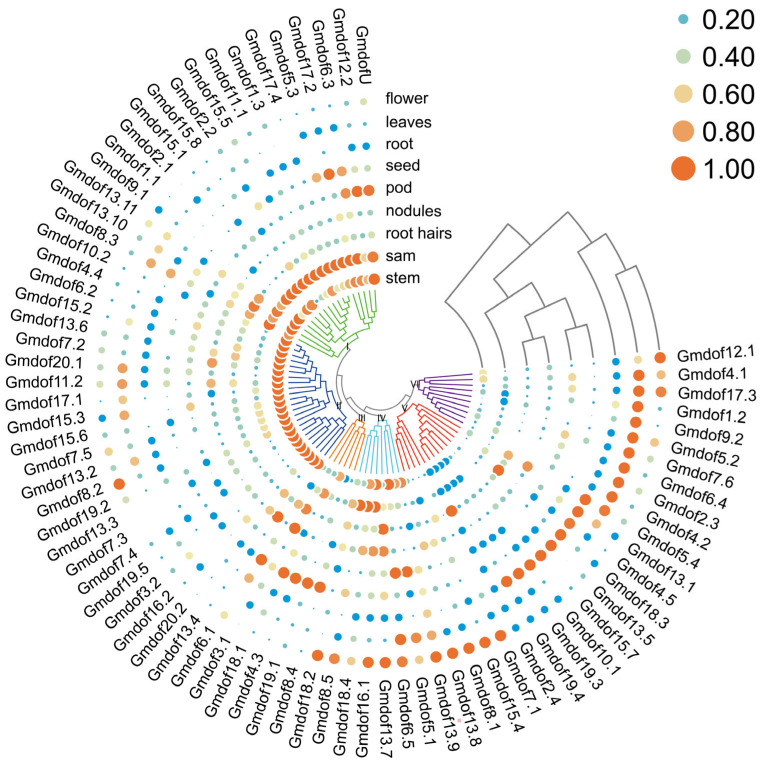
Expression patterns of 79 *Dof* genes in nine tissues of soybean.

To further understand the biological functions of the *Gmdof* genes in soybean, we analyzed the expression data of *Gmdof* genes in various tissues, including flowers, leaves, roots, seeds, pods, nodules, root hairs, apical meristems, and stems (Figure 6). The expression profiles of genes within each group exhibited similar patterns across different tissues. For instance, the 18 *Gmdof* genes in group I showed relatively high expression in the apical meristem and stem, with comparatively lower expression in other tissues. In group II, all 18 *Gmdof* genes exhibited high expression in the stem, similar to group I, while the expression in other tissues was relatively low or nearly undetectable. In group V, nine *Gmdof* genes had higher expression in root. Notably, the *Gmdof* genes in group VI, which were highly similar to the Arabidopsis Dof genes *AtDof5.5* (*CDF1*), *AtDof5.2* (*CDF2*), and *AtDof3.3* (*CDF3*), showed high expression in the leaves, which suggested that these genes may be involved in regulating the photoperiodic flowering pathway in soybean, similar to their counterparts in Arabidopsis. In summary, most *Dof* family genes exhibit relatively high expression levels in specific tissues, while maintaining low expression in other tissues, showing distinct tissue-specific expression characteristics.

### 2.7. Expression Pattern of Some Dof Genes in Response to Salt

**Figure 7:**
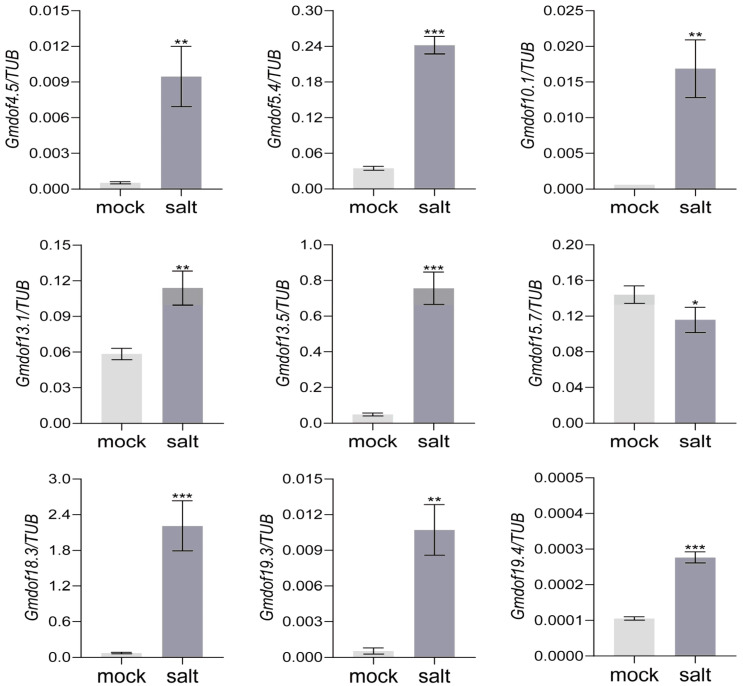
Expression pattern of some *Gmdof* genes in response to salt. * means *p* ≤ 0.05, ** means *p* ≤ 0.01, and *** means *p* ≤ 0.001. Mork represents blank control with deionized water. Salt represents 200 μM NaCl.

Based on the expression patterns of the Gmdof family in various tissues, nine Gmdof (*Gmdof4.5*, *Gmdof5.4*, *Gmdof10.1*, *Gmdof13.1*, *Gmdof13.5*, *Gmdof15.7*, *Gmdof18.3*, *Gmdof19.3*, *Gmdof19.4*) in group V were identified with higher expression in root. To investigate their regulatory roles, soybean at V1 stage were treated with 200 μM NaCl to identify the expression patterns of *Gmdof* genes response to salt. The result showed that nine genes all responded to salt stress significantly (Figure 7). After salt stress, the expression level of *Gmdof15.7* significantly decreased and negatively regulated salt tolerance in soybeans, while the expression levels of the other eight genes significantly increased, indicating that these genes positively regulate salt tolerance in soybeans. All these nine genes provide genetic resources for new varieties of salt-tolerant soybeans in molecular breeding.

## 3. Discussion

With the development of integrated genomics and the ability to acquire large-scale soybean genome and multi-omics datasets, there are now opportunities to effectively predict phenotypes and explore gene functions [27]. The Dof (DNA-binding with one finger) transcription factor, a classic protein within the Cys2-His2 zinc finger (ZF) superfamily [28], plays a crucial role in several vital processes in plants. However, research on this gene in soybean, and even in leguminous plants in general, remains limited. This study utilized publicly available soybean genome data to conduct a comprehensive genome-wide identification and analysis of the soybean *Dof* gene family.

In this study, through extensive and comprehensive analysis, numerous members of the *Dof* gene family were identified within the soybean genome. Genome-wide analysis revealed the presence of 79 full-length *Dof* genes. Furthermore, phylogenetic analysis of soybean Dof proteins with those from Arabidopsis, rice, maize, and alfalfa revealed five major homologous groups. As most Arabidopsis Dof genes with similar functions tend to cluster within the same subgroups, it is likely that the *GmDof* genes within the same subgroups also share similar functions. *AtDof2.4* and *AtDof5.8* might function in the early but different processes for vascular development [29]. In this study, phylogenetic analysis revealed that *Gmdof11.2* showed significant similarity with *Atdof2.4*, while *Gmdof2.1* and *Gmdof16.2* showed significant similarity with *AtDof5.8*. Importantly, these three *Gmdofs* genes had the highest expression in the stems, indicating that they may also be involved in the vascular development of soybeans. Meanwhile, AtDOF5.8 protein was the upstream regulator of *AtNAC069*, which integrated auxin and salt signals to regulate *Arabidopsis thaliana* seed germination [16]. *Gmdof2.1* and *Gmdof16.2* was responsive to abiotic stress. In group 4, some *GmDof* genes exhibited significant similarity to Arabidopsis Dof genes *AtDof5.5* (*CDF1*), *AtDof5.2* (*CDF2*), *AtDof3.3* (*CDF3*), and *AtDof1.7* (*CDF5*), which participated in the Arabidopsis photoperiodic flowering pathway by inhibiting the transcription of the *CONSTANS* gene [30]. Interestingly, among these *Gmdof* genes, *Gmdof5.2*, *Gmdof17.3* and *Gmdof8.1* had higher expression in flower, indicating their functions in the photoperiodic flowering pathway of soybean. In addition, four soybean Dof genes (*Gmdof4.1*, *Gmdof6.5*, *Gmdof17.1*, *Gmdof5.1*) clustered with the Arabidopsis Dof gene *AtDof5.4*, the overexpression of which resulted in dwarfing of Arabidopsis plants by reducing cell size and number [31]. Importantly, *Gmdof6.5*, *Gmdof17.1* and *Gmdof5.1* had high expression in stem, indicating that they may also be involved in the plant height of soybeans.

Soybean has undergone two whole-genome duplications during its evolutionary history, which has allowed the plant to acquire new genes and gain genetic diversity [32,33]. Among the 79 Gmdof genes in soybean, collinearity analysis showed that a total of 284 gene duplication segments were identified, indicating *Gmdof* genes provided strong evidence for the whole-genome duplication events in soybean. One relevant study that explores evolutionary dynamics of duplicated genes is the analysis of the *NOX* gene family in soybean [34]. This study identified 17 *NOX* genes, with 8 duplicated gene pairs formed by a whole-genome duplication event approximately 13 million years ago [34]. Similarly, the study of the APETALA2/Ethylene-Responsive transcription factors (AP2/ERFs) in sorghum reveals insights into gene family evolution and redundancy [35]. This redundancy is thought to provide a buffer against environmental stresses, allowing for flexible responses to changing conditions [34,35].

Furthermore, the study of organ and cell type-specific complementary expression patterns in *Arabidopsis thaliana* provides additional insights into the regulatory neofunctionalization of duplicated genes. The research found that a significant proportion of whole-genome and tandem duplicate pairs show reciprocal expression patterns, where only one copy is expressed in certain organ types, while the other is expressed in different tissues [36]. This pattern is indicative of regulatory subfunctionalization or neofunctionalization, allowing for the retention and divergence of duplicated genes [36]. In this study, most of the Gmdof proteins were located in the nucleus, with only six Gmdofs localized outside the nucleus. Importantly, Gmdof8.1 and Gmdof5.4 were homologous, but located in the cytoplasm and the nucleus, respectively. This pattern is similar to that observed for Gmdof11.2 (plasma membrane), Gmdof12.1 (nucleus), Gmdof6.1 (chloroplast), Gmdof4.5 (nucleus), Gmdof19.3 (nucleus), and Gmdof19.4 (chloroplast). These homologous Gmdof genes located in different organs may have gene subfunctionalization. These studies collectively illustrate the complexity of gene duplication and expression evolution in plants, emphasizing the roles of subfunctionalization and neofunctionalization in shaping the functional landscape of duplicated genes.

In this study, the expression profiles of genes within each group exhibited similar patterns across different tissues. Most *Dof* family genes exhibit relatively high expression levels in specific tissues, while maintaining low expression in other tissues, showing distinct tissue-specific expression characteristics, which is a common feature observed in various gene families across different species. Nine *Gmdof* genes with high expression in root were identified to response to salt stress. Interestingly, these genes contained at least one ABA response element by the promoter cis-acting elements analysis of Gmdof gene family. The ABA signaling pathway generally encompasses the initial stress signal perception, cellular signal transduction, and regulation of genes expressions related to ABA biosynthesis and catabolism, which in turn regulate the accumulation of ABA and trigger salt stress responses [37]. These findings indicated these nine *Gmdof* genes could be involved in salt stress by ABA signaling pathway.

## 4. Materials and Methods

### 4.1. Database Search and Sequence Retrieval

We obtained the protein and genome sequence files for soybean, *Arabidopsis thaliana*, *Medicago truncatula*, *Oryza sativa*, and maize from the Plant Genome Database (Phytozome, https://phytozome-next.jgi.doe.gov/, accessed on 4 July 2023) [38]. The Dof genes in soybean reported in previous studies [25] were extracted using TBtools-II v2.210 (https://github.com/CJ-Chen/TBtools/releases, accessed on 6 July 2023) [39].

To identify the Dof genes in soybean, two approaches were employed. First, we retrieved the conserved domain zinc finger, Dof-type (PF02071), from the PFAM database (http://pfam.xfam.org, accessed on 10 July 2023) for the model plant *Arabidopsis thaliana* and used it as a query sequence. We then performed a search for candidate BPS protein sequences across four species using Hmmer 3.0 (http://hmmer.org, accessed on 10 July 2023) with default parameters. Second, previously reported Dof proteins in soybean were used as query sequences, and a BLASTP search was conducted within the soybean protein sequence file using BLAST 2.5.0 for proteins (default parameters, e-value < 1 × 10^−5^). The resulting alignments were merged and redundant sequences were removed. To confirm the presence of the zinc finger domain in each sequence, further verification was carried out using SMART (http://smart.embl-heidelberg.de/, accessed on 10 July 2023). Subcellular localization of the Dof genes was predicted using the BUSCA server (http://busca.biocomp.unibo.it/, accessed on 15 July 2023) [40]. Additionally, the physicochemical properties of the Dof proteins (e.g., amino acid count, molecular weight, and isoelectric point) were analyzed using the ProtParam tool (http://web.expasy.org/compute_pi/, accessed on 15 July 2023) [41].

### 4.2. Chromosome Mapping, Synteny and Phylogenetic Analysis

The chromosomal mapping of all identified *Dof* genes in soybean were visualized using TBtools-II v2.210 software. TBtools was also utilized for synteny analysis and Ka/Ks analysis. Full-length amino acid sequences from soybean, *Arabidopsis thaliana*, *Medicago truncatula*, *Oryza sativa*, and maize were aligned using MUSCLE (default parameters) in MEGA7 [42]. Subsequently, a phylogenetic tree was constructed using the ML model in MEGA7, and further modifications and optimization were performed using iTOL (https://itol.embl.de, accessed on 10 August 2023).

### 4.3. Gene Structure and Conversed Motif Analysis

The gene structure of *Dof* genes was analyzed using the Gene Structure Display Server (GSDS, http://gsds.cbi.pku.edu.cn/, accessed on 10 August 2023) [43]. Conserved motifs were identified using the MEME tool (http://meme-suite.org/tools/meme, accessed on 10 August 2023) [44]. The results of gene structure and motif analyses were then integrated and visualized using TBtools.

### 4.4. Collinearity and Cis-Regulatory Element Analysis

First, the Fasta Stats tool in TBtools-II v2.210 was used to analyze the soybean genome sequence to calculate the length of each chromosome. Next, the Gene Density Profile tool in TBtools-II was used to process the gene annotation file and obtain gene density data. Subsequently, the Blast Compare Two Seqs tool in TBtools-II v2.210 was employed to align the soybean genome sequence with itself. Gene position information was extracted using the TBtools-II v2.210. Finally, the results were visualized and decorated using the Advanced Circos tool in TBtools-II v2.210. For cis-regulatory element analysis, 2000 bp sequences upstream from the initiation codon of the putative *Gmdofs* were retrieved. These sequences were subjected to search in the PlantCARE database (https://bioinformatics.psb.ugent.be/webtools/plantcare/html/, accessed on 17 March 2025) [45] to identify cis-regulatory elements.

### 4.5. Expression Pattern of Gmdofs

The expression values of Gmdofs (FPKMs) were retrieved from the Phytozome website (https://phytozome-next.jgi.doe.gov/, accessed on 24 August 2023). We analyzed the differential expression of *Gmdofs* across different tissues, including flowers, leaves, roots, seed, pod, nodules, root hair, sam, and stems. The expression values of *Gmdofs* were normalized to log2 (FPKM + 1), followed by a 0–1 standardization, and HeatMap in TBtools was used for visual drawing.

Williams 82 soybean seedlings at V1 stage were treated with 200 µM NaCl in a short-day incubator (8 h light/16 h dark, 25 °C). A blank control consisted of deionized water. After treatment, the seedling’s root were collected at 9 h after treatment, and they were promptly frozen in liquid nitrogen. For every sample, 3 biological replicates were collected. Total RNA was extracted by using the Plant RNA Extraction Kit (TaKaRa, Tokyo, Japan). The total amount of RNA (approximately 2 µg) was used to produce cDNA by using the PrimeScriptTM II 1st Strand cDNA Synthesis Kit (TakaRa, Japan). The 2× Taq Pro Universal SYBR qPCR Master Mix (Vazyme, Nanjing, China) was utilized for qRT-PCR on the Roche LightCycler^®^ 480 II. The PCR amplification system comprised a total volume of 20 µL, including 2 µL template DNA, 10 µL 2× mix, and 0.8 µL of both forward and reverse primer (1 µM). The remaining volume was adjusted to 20 µL with ddH_2_O. The PCR amplification conditions were as follows: initial denaturing at 95 °C for 1 min, followed by 45 cycles for 95 °C for 5 s, 60 °C for 20 s, and then melting the curve starting at 95 °C for 15 s, followed by 60 °C for 1 min, and finally at 95 °C for 15 s. The 2^−ΔΔCt^ method was used to calculate the relative expression levels. Each treatment was replicated three biological times and three technical times. Statistical significance was determined using Student’s *t*-tests in GraphPad Prism 8, with the aim to identify significant differences. The qRT-PCR primers were designed by Primer3 v4.1.0 (Supplementary Table S2).

## 5. Conclusions

In this study, through extensive and comprehensive analysis, genome-wide analysis revealed the presence of 79 full-length *Dof* genes, non-randomly distributed across 19 chromosomes. Phylogenetic analysis of soybean Dof proteins with those from Arabidopsis, rice, maize, and alfalfa revealed five major homologous groups. Soybean intraspecific collinearity analysis also provided strong evidence for soybean whole-genome duplication. Global expression profile analysis provided insights into the functional differentiation of soybean *Dof* gene family members, with most *Gmdof* genes exhibiting specific spatiotemporal expression patterns, and nine Gmdof genes with higher expression in root were identified response to salt stress significantly by qRT-PCR. Finally, the possible biological function of *Gmdof11.2*, *Gmdof2.1*, *GmDof16.2*, and so on were explored based on phylogenetic tree and expression pattern analysis. These analysis contribute to the subsequent study of the functional properties of the *Dof* gene, which is beneficial for the various vital processes of soybean.

## Data Availability

The original contributions presented in this study are included in the article/Supplementary Materials. Further inquiries can be directed to the corresponding author(s).

## Supplementary Materials

The supporting information can be downloaded at https://www.mdpi.com/article/10.3390/ijms26136192/s1.
