# Supplementary material for: Genome-Wide Analysis of the DNA-Binding with One Finger Gene Family Reveals Soybean Expression Pattern and Functional Analysis
Source: Int J Mol Sci. 2025 Jun 27;26(13):6192. doi: 10.3390/ijms26136192 (PMC12250066; doi:10.3390/ijms26136192)
Supplement: Supplementary file 1 [file ijms-26-06192-s001.zip › Supplementary Table S2-Real-time PCR primers information for quantitative analysis..pdf]

Supplementary Table S2 Real-time PCR primers information for quantitative analysis.

**Primer Name**	**Primer sequence**
Gmdof4.5-RT-F	AACACCAAAGCCATCAACGC
Gmdof4.5-RT-R	AATTTGGCGAACACGAGAGC
Gmdof5.4-RT-F	TCAGGAGGAGAGGTTGGCTT
Gmdof5.4-RT-R	CTTTGACTTTCATGCTGAGCTGA
Gmdof10.1-RT-F	TCCTGAACAACAACCACAAACAC
Gmdof10.1-RT-R	TCATAGCACCACCACCATGA
Gmdof13.1-RT-F	TGGGGATGGTGAAGAACACG
Gmdof13.1-RT-R	CTCCTTCTCGGTAGAGGGGT
Gmdof13.5-RT-F	GATGGGTCAGGGACACGTTG
Gmdof13.5-RT-R	CCTTGATGCTGAGCTGAGGT
Gmdof15.7-RT-F	ATGGGCGAGGAATCTCAAGG
Gmdof15.7-RT-R	GCATCTCGGGCAGGGTATTA
Gmdof18.3-RT-F	CCCCCACCCAATCATCAGAA
Gmdof18.3-RT-R	CGGTGGACAGAGCAACAGAT
Gmdof19.3-RT-F	CGGTGAGAGTATCCAAATG
Gmdof19.3-RT-R	CACCAGGCAACGGTGGCAAC
Gmdof19.4-RT-F	GGAGAAATGGTGGAAGGCTAA
Gmdof19.4-RT-R	GCAGCCCTTGCAAAAGTACC
